# A Case of Acute Hypertriglyceridemia-Induced Pancreatitis in Pregnancy and Its Clinical Implications

**DOI:** 10.1155/2024/5896861

**Published:** 2024-10-10

**Authors:** Corley Rachelle Price, Anthony Kendle, Mary Ashley Cain

**Affiliations:** Department of Obstetrics and Gynecology, University of South Florida Morsani College of Medicine, Tampa, Florida, USA

**Keywords:** acute pancreatitis, diabetic ketoacidosis, hypertriglyceridemia, pregnancy

## Abstract

Acute hypertriglyceridemia-induced pancreatitis (HTGP) is an uncommon occurrence during pregnancy. Prompt diagnosis and initiation of treatment are indicated to prevent adverse maternal and neonatal outcomes. We present the case of a pregnant female who was diagnosed with HTGP at 34 weeks gestation and subsequently developed diabetic ketoacidosis (DKA) and preeclampsia with severe features. We describe the pathophysiology of acute HTGP and its relation to the gravid state and review available treatment options though data remains limited. Our case emphasizes the potential sequelae of HTGP in pregnancy, the need for a multidisciplinary approach for optimal care, and the importance of early treatment in improving maternal and neonatal outcomes.

## 1. Introduction

Acute pancreatitis is a rare but serious complication of pregnancy that can lead to significant perinatal morbidity and mortality. Studies have demonstrated an increased risk for intensive care unit (ICU) admission, metabolic disturbances, hypovolemia, sepsis, preterm delivery, fetal distress, and fetal demise among patients with acute pancreatitis during pregnancy [1, 2]. Delays in diagnosis and treatment may increase the risk for pregnancy-related complications. We present a case of a primigravid patient with acute hypertriglyceridemia-induced pancreatitis (HTGP) and subsequent development of diabetic ketoacidosis (DKA) at 34 weeks gestation.

## 2. Case Report

A 38-year-old primigravid Hispanic female presented with nausea, vomiting, and epigastric pain at 34 weeks and 4 days gestation. Her pregnancy was complicated by chronic hypertension, suspected type 2 diabetes mellitus due to an elevated 3-h glucose tolerance test (GTT) per Carpenter and Coustan criteria at 16 weeks gestation [3], and obesity with a body mass index (BMI) of 35.5 kg/m^2^. Her known family history was limited. Upon presentation, she received intravenous fluids, intravenous morphine, and antiemetics with minimal symptomatic improvement. Her vital signs demonstrated mild range blood pressure and were otherwise unremarkable. Upon initial blood draw, the physicians and nursing staff noted that her blood sample was lipemic in appearance. The patient's initial laboratory results with reference values during pregnancy are shown in Table 1. Gastroenterology was consulted, and magnetic resonance cholangiopancreatography (MRCP) was performed. MRCP demonstrated severe acute interstitial pancreatitis without evidence of choledocholithiasis. Laboratory and examination findings were consistent with HTGP for which fluid resuscitation and analgesia with intravenous morphine were continued. The patient underwent continuous fetal monitoring and was noted to have a category I tracing.

**Figure 1:**
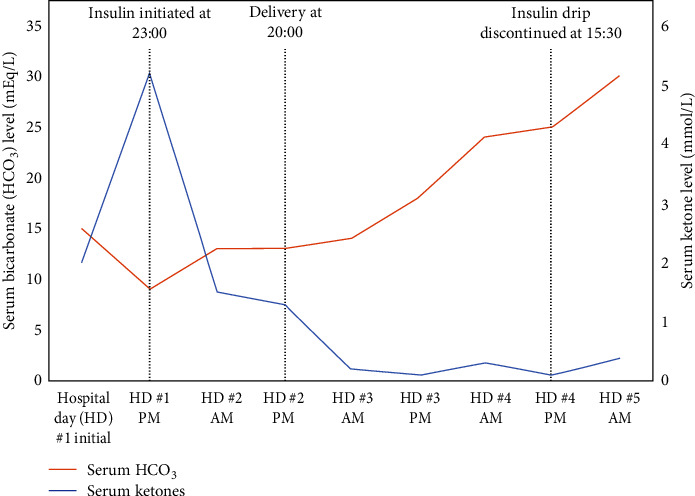
Trend in serum bicarbonate and serum ketones with a continuous insulin infusion for treatment of diabetic ketoacidosis.

**Figure 2:**
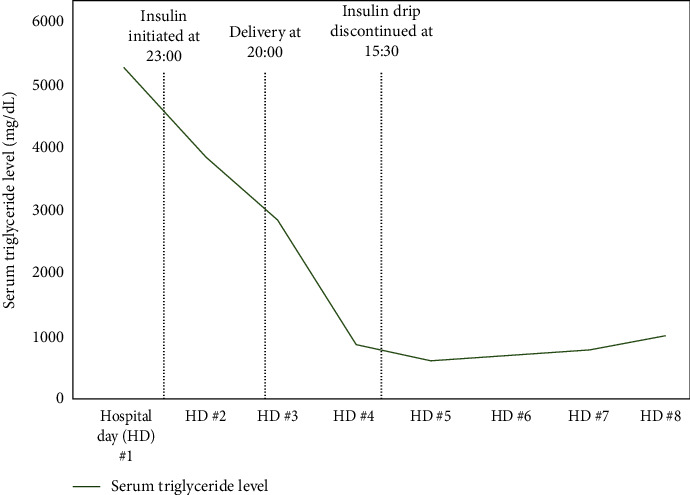
Trend in triglyceride level with a continuous insulin infusion for treatment of hypertriglyceridemia-induced acute pancreatitis.

Eleven hours later, the patient was noted to have worsening metabolic acidosis with arterial blood gas (ABG) showing pH 7.27 and HCO_3_ 9 mEq/L. Her anion gap increased to 16 mEq/L, and her serum ketone level increased to 5.2 mmol/L (Figure 1). The patient's blood glucose level remained stable at 135 mg/dL. DKA was diagnosed based on worsening anion gap metabolic acidosis and ketonemia. The patient was admitted to the ICU on a continuous insulin infusion. Endocrinology and critical care were consulted. Continuous fetal monitoring was consistent with a category II tracing. The patient's mental status remained appropriate throughout this time. On hospital day 2, the patient's triglyceride level decreased to 4659 mg/dL (Figure 2), and her anion gap metabolic acidosis improved with ABG showing pH 7.42 and HCO_3_ 13 mEq/L, serum ketones 1.5 mmol/L, and anion gap 12 mEq/L (Figure 1). The patient was transferred from the ICU to the labor and delivery unit for continuous monitoring. Later that day, the patient met criteria for preeclampsia with severe features based on severe range blood pressure, and she was started on magnesium sulfate for seizure prophylaxis. The decision was made to proceed with delivery via a primary cesarean section under general anesthesia given concern for prolonged labor induction in a primiparous pregnancy. The patient received general anesthesia rather than neuraxial anesthesia as she had received prophylactic enoxaparin several hours prior to proceeding with delivery. Delivery resulted in a healthy newborn male weighing 3645 g with Apgar scores 8 and 9 at 1 min and 5 min, respectively. Umbilical cord blood gas showed pH 7.14, base deficit 9 mEq/L, and HCO_3_ 22 mEq/L. The total estimated intraoperative blood loss was 600 cc.

Following delivery, the patient was admitted to the ICU for further management as she remained intubated in the immediate postoperative period. She was continued on magnesium sulfate for seizure prophylaxis and a continuous insulin infusion for treatment of hypertriglyceridemia. By hospital day 3, her anion gap metabolic acidosis had resolved, and her serum ketone level had normalized (Figure 1). Her postoperative course was complicated by the need for blood transfusion, transient hypotension requiring vasopressors, and acute kidney injury (AKI) that was prerenal in etiology. Nephrology was consulted, and the patient underwent diuresis using bumetanide and metolazone due to concern for hypervolemia and third spacing. Her AKI resolved following diuresis with normalization of her creatinine level by hospital day 4. The continuous insulin infusion was discontinued once the patient's triglyceride level reached ~500 mg/dL on hospital day 4 (Figure 2). The patient was extubated without complications on hospital day 5 and transferred to the postpartum floor on hospital day 7. She experienced multiple severe range blood pressures requiring acute treatment, and nifedipine 30 mg BID was started to maintain blood pressure control. The patient was counseled on a strict low-fat diet by the dietician team and was started on atorvastatin, omega-3 acid, and fenofibrate for the management of hypertriglyceridemia. She was administered subcutaneous insulin for glycemic control and was transitioned to metformin 1000 mg BID as well as nighttime and sliding-scale mealtime insulin. The patient was discharged in stable condition on hospital day 9.

At the 6-week postpartum visit, the patient was recovering well. She was evaluated by internal medicine and was noted to have an improved Hgb A1c 6.1% (compared to Hgb A1c 7.4% at 28 weeks gestation), triglyceride level of 588 mg/dL, total cholesterol level of 349 mg/dL, and LDL cholesterol level of 189 mg/dL. She reported compliance with metformin 1000 mg BID for glycemic control and atorvastatin, omega-3 acid, and fenofibrate for hypercholesterolemia. The patient was encouraged to continue nifedipine 30 mg BID due to persistent hypertension. Although the etiology of the patient's severe hypertriglyceridemia was not determined during her pregnancy, she had outpatient endocrinology follow-up scheduled for additional evaluation and management.

## 3. Discussion

Acute pancreatitis is an uncommon occurrence in pregnancy with most studies reporting one case per 1000–10,000 pregnancies [1, 4, 5]. Gallstones remain the most common cause of acute pancreatitis in pregnancy, followed by alcohol abuse and hypertriglyceridemia [1, 5, 6]. Although maternal and fetal mortality rates were previously reported as high as 7% and 60%, respectively, the maternal and fetal mortality rates have significantly declined to 0% and 3%, respectively [5]. Acute pancreatitis continues to be associated with adverse outcomes including preterm delivery, fetal demise, and adverse neonatal outcomes such as stillbirth, prematurity, structural anomalies, and low birthweight [6]. Due to the potential morbidity of acute pancreatitis in pregnancy, prompt diagnosis and treatment are essential in reducing risk for both the mother and fetus.

Acute pancreatitis is diagnosed using the revised Atlanta classification system which requires that two or more of the following criteria be met: (1) abdominal pain suggestive of pancreatitis, (2) serum amylase or lipase levels greater than three times the upper limit of normal, and (3) characteristic imaging findings on either computed tomography with contrast or magnetic resonance imaging [7]. The patient in our case study met criteria for acute pancreatitis based on her epigastric pain and elevated amylase and lipase levels of 553 U/L and 1728 U/L, respectively. Given her elevated triglyceride level at 5246 mg/dL and the absence of biliary obstruction on imaging studies, the patient was diagnosed with severe HTGP.

Physiological increases in triglyceride and total cholesterol levels are expected during pregnancy as these metabolites are needed to support the demands of the placenta and growing fetus, with the serum triglyceride level peaking during the third trimester at a value two- to fourfold greater than prepregnancy values [1, 4, 5]. HTGP is typically associated with serum triglyceride levels of >1000 mg/dL [4], which is well above the expected physiologic increase in triglyceride levels. The exact pathophysiology of acute pancreatitis secondary to hypertriglyceridemia involves several mechanisms. One mechanism involves hydrolysis of triglycerides by pancreatic lipase to produce free fatty acids. These free fatty acids accumulate in the pancreas and cause injury to the acinar cells and pancreatic capillaries, which can ultimately lead to pancreatic ischemia and acidosis [8]. Another mechanism suggests that elevated levels of chylomicrons in pancreatic capillaries increase blood viscosity, thus resulting in pancreatic ischemia [8]. Regardless of mechanism, pancreatic inflammation can induce beta cell dysfunction, resulting in transient insulin deficiency. This insulin deficiency can precipitate or worsen DKA in pregnant individuals, as observed in our case study [9].

The interplay between HTGP and DKA is complex given their overlapping processes. These two conditions may occur concurrently, making it difficult to determine the inciting event. DKA due to insulin resistance or insulin deficiency can result in increased lipolysis of adipose tissue, increased free fatty acid release and very low-density lipoprotein (VLDL) production, and decreased lipoprotein lipase activity [10]. The accumulation of free fatty acids can cause acinar cell damage leading to pancreatitis as mentioned previously [8, 10]. Conversely, HTGP can trigger DKA through *β*-cell dysfunction and insulin deficiency [10]. Although studies have attributed HTGP to be a result of DKA, fewer studies identify HTGP as the inciting event [10, 11]. The patient in our case study initially presented with mild metabolic acidosis with ketonemia and significantly elevated triglyceride levels suggesting HTGP (Table 1). Her clinical status later deteriorated with intravenous fluid resuscitation, and she was noted to have a worsening anion gap metabolic acidosis and ketonemia suggesting subsequent or worsening DKA. The intricate relationship between these two conditions demonstrates that both require prompt recognition and management to prevent continued clinical deterioration.

An association between HTGP and the development of preeclampsia with severe features also exists. The patient in our case study had multiple risk factors for developing preeclampsia including nulliparity, chronic hypertension, suspected pregestational diabetes mellitus, advanced maternal age >35 years old, and obesity [12]. Studies have also demonstrated an association between maternal hypertriglyceridemia and increased risk for preeclampsia [13, 14]. The pathophysiology behind this association is complex and could involve increased oxidative stress resulting in endothelial dysfunction, insulin resistance causing decreased lipoprotein lipase activity and increased triglyceride levels, and/or increased lipolytic activity resulting in increased free fatty acid uptake by endothelial cells and formation of triglycerides [14, 15]. In our case study, the patient met diagnostic criteria for superimposed preeclampsia with severe features based on severe range blood pressures requiring acute treatment. These elevated blood pressures were an acute change compared to her baseline blood pressures from chronic hypertension. Although persistent epigastric pain unresponsive to analgesia can be a component of preeclampsia with severe features [12], the patient's epigastric pain was attributed to her acute pancreatitis episode rather than to preeclampsia with severe features. The development of superimposed preeclampsia with severe features in our patient was likely multifactorial given her numerous risk factors. Although her acute pancreatitis episode likely contributed to the development of superimposed preeclampsia, it was not the sole causative factor.

Given the rare occurrence of HTGP during pregnancy, there are no established treatment guidelines specific to pregnant individuals. Anecdotal evidence and preferred treatments for nonpregnant individuals are typically used which can include supportive management, insulin therapy, heparin, or plasmapheresis [15]. Initial supportive management should include aggressive fluid resuscitation, analgesia, and diet restriction [16]. Insulin infusion can be considered even when an individual remains euglycemic as it is effective in reducing serum triglyceride levels by enhancing lipoprotein lipase activity [4, 17]. Heparin alone or in combination with insulin therapy has also been shown to decrease triglyceride levels by releasing lipoprotein lipase from endothelial cells thus accelerating its enzymatic activity, however its use in pregnancy may be limited by potential bleeding risk if delivery is anticipated for maternal or fetal indications [17–19]. The use of plasmapheresis for refractory cases of HTGP remains a category III indication as per the American Society for Apheresis (ASFA) guidelines as data regarding its use is limited to observational studies and case reports [20]. Its use in pregnancy is similarly confined to case studies with varying pregnancy outcomes [19]. Plasmapheresis decreases triglyceride levels by filtering an individual's blood, removing triglyceride particles and inflammatory mediators, and returning the filtered blood with replacement fluid to the individual [1, 4, 19]. Plasmapheresis can rapidly reduce triglyceride levels, however it can have potential adverse pregnancy effects including hypotension, decreased placental blood flow, nonreassuring fetal status, emergent cesarean delivery, coagulopathies, and cardiac arrhythmias [1].

When comparing the effectiveness of plasmapheresis and conservative management in reducing triglyceride levels, studies in nonpregnant populations have shown varied results. In a study by Dichtwald et al. [21], plasmapheresis was associated with prolonged admission, need for ventilation, and need for inotropic support and dialysis, while no significant difference was noted in the mortality rate or rate of decline in serum triglyceride levels when compared with individuals undergoing conservative management (which included fasting, intravenous fluid resuscitation, and insulin therapy) [21]. Another study showed a more rapid decline in serum triglyceride levels over a 48-h period when plasmapheresis was used. However, the decline in serum triglyceride levels was equivocal after 72 h regardless of whether plasmapheresis or conservative management was used [22]. These studies demonstrate that the rate of decline in triglyceride levels with plasmapheresis can vary according to the protocol and technique, but overall, conservative management and plasmapheresis are both effective treatment modalities. In pregnancy, plasmapheresis can be considered in refractory cases of HTGP, however maternal status, gestational age, timing of delivery, and fetal status must be considered prior to initiation due to the potential for maternal and fetal morbidity. The patient in our case study initially received supportive management including fluid resuscitation, analgesia, and diet restriction without improvement and thus, was started on a continuous insulin infusion. Although plasmapheresis was strongly considered in our case, the patient's serum triglyceride level and acid–base status significantly improved with insulin therapy by hospital day 2 and plasmapheresis was not needed.

## 4. Conclusion

HTGP is a rare, but serious, medical condition during pregnancy. Prompt recognition and treatment are essential for optimizing maternal and neonatal outcomes. Data regarding treatment options for HTGP are limited to case studies given its rare occurrence but can include supportive management, insulin therapy, heparin, or plasmapheresis depending on case severity. Our case highlights potential sequelae of HTGP in pregnancy such as DKA, preeclampsia, preterm delivery, and need for ICU admission. The association between HTGP and subsequent DKA is not well documented but provides an interesting and dynamic perspective on the complex physiology in pregnancy. Our case also emphasizes the importance of a multidisciplinary approach to patient care which included specialists from maternal–fetal medicine, critical care, gastroenterology, endocrinology, anesthesia, nutrition, and nursing. With the expertise of multiple specialists, the patient had a successful preterm delivery of a healthy newborn and was discharged in stable condition.

## Figures and Tables

**Table 1:** Patient's initial laboratory results on presentation.

Laboratory parameter	Initial patient value	Reference range
White blood cell count	7.70 × 10^3^/uL	4.6–10.2 × 10^3^/uL
Hemoglobin	10.1 g/dL	12.2–16.2 g/dL
Hematocrit	28.8%	37.7%–47.9%
Platelets	255 × 10^3^/uL	142–424 × 10^3^/uL
Sodium	124 mEq/L	135–148 mEq/L
Potassium	4.7 mEq/L	3.5–5.3 mEq/L
Chloride	95 mEq/L	98–107 mEq/L
BUN	9 mg/dL	6–20 mg/dL
Creatinine	0.6 mg/dL	0.57–1.11 mg/dL
AST (SGOT)	45 U/L	5.0–34 U/L
ALT (SGPT)	15 U/L	5.0–55 U/L
Total bilirubin	0.3 mg/dL	0.1–1.2 mg/dL
Alkaline phosphatase	168 U/L	40–150 U/L
Glucose	108 mg/dL	70–110 mg/dL
Anion gap	14 mEq/L	5–13 mEq/L
Lipase	1728 U/L	4–78 U/L
Amylase	553 U/L	25–125 U/L
Triglycerides	5246 mg/dL	<150 mg/dL
Total cholesterol	1247 mg/dL	<200 mg/dL
Ketones	2.0 mmol/L	0–0.3 mmol/L
Latic acid	1.5 mmol/L	0.17–2.2 mmol/L
Hemoglobin A1c	7.4%	<5.7%
Arterial blood gas (ABG)		
pH	7.38	7.40–7.46
pCO_2_	25 mmHg	26–32 mmHg
pO_2_	105 mmHg	101–106 mmHg
HCO_3_	15 mEq/L	18–21 mEq/L

## Data Availability

The authors have nothing to report.
